# Supplementary material for: The interactome of intact mitochondria by cross-linking mass spectrometry provides evidence for coexisting respiratory supercomplexes
Source: Mol Cell Proteomics. 2017 Dec 8;17(2):216–32. doi: 10.1074/mcp.RA117.000470 (PMC5795388; doi:10.1074/mcp.RA117.000470)
Supplement: Supplemental Data [file supp_RA117.000470_133922_0_supp_23978_fzffwf.zip › spectra_annotation/mito_DR_spectra_annotation/141-1-5-1-3-1.pdf]

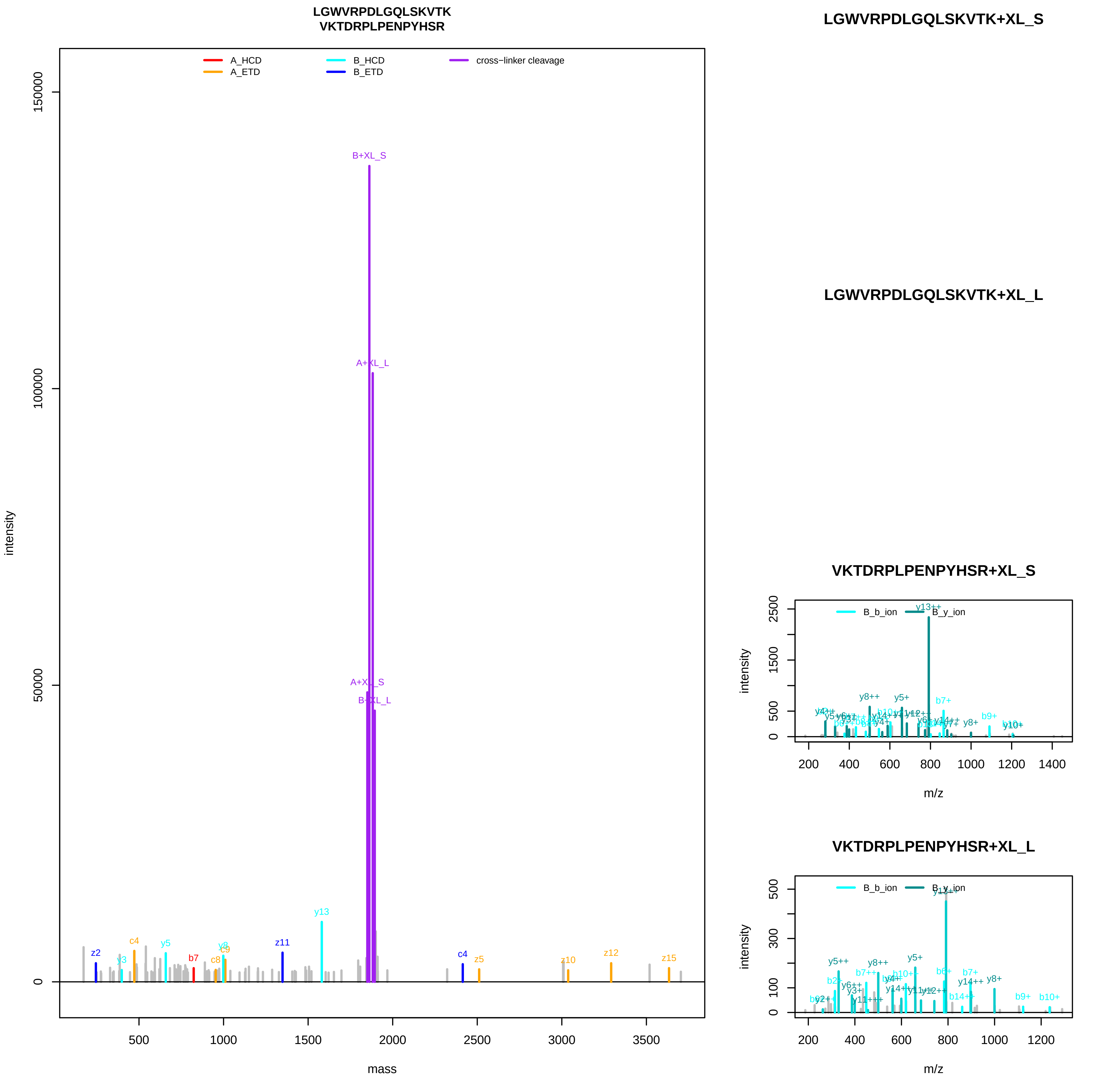

LGWVRPDLGQLSKVTK
VKTDRPLPENPYHSR
A_HCD
A_ETD
B_HCD
B_ETD
cross−linker cleavage
B+XL_S
A+XL_L
A+XL_S
B+XL_L
intensity
mass
z2
y3
c4
y5
b7
c8
y8
c9
z11
y13
c4
z5
z10
z12
z15
LGWVRPDLGQLSKVTK+XL_S
LGWVRPDLGQLSKVTK+XL_L
VKTDRPLPENPYHSR+XL_S
B_b_ion
B_y_ion
y13++
y8++
y5+
b7+
y8+
b9+
y10+
m/z
VKTDRPLPENPYHSR+XL_L
y5++
y8++
y5+
b6+
b7+
y14++
y8+
b9+
b10+
